# Insight into the incredible effects of microwave heating: Driving changes in the structure, properties and functions of macromolecular nutrients in novel food

**DOI:** 10.3389/fnut.2022.941527

**Published:** 2022-10-13

**Authors:** Xuan Deng, Haozhou Huang, Shengjie Huang, Ming Yang, Jing Wu, Zhimin Ci, Yanan He, Zhenfeng Wu, Li Han, Dingkun Zhang

**Affiliations:** ^1^State Key Laboratory of Southwestern Chinese Medicine Resources, Pharmacy School, Chengdu University of Traditional Chinese Medicine, Chengdu, China; ^2^Key Laboratory of Modern Preparation of Chinese Medicine, Ministry of Education, Jiangxi University of Traditional Chinese Medicine, Nanchang, China; ^3^State Key Laboratory of Innovation Medicine and High Efficiency and Energy Saving Pharmaceutical Equipment, Jiangxi University of Traditional Chinese Medicine, Nanchang, China; ^4^Xinqi Microwave Co., Ltd., Guiyang, China

**Keywords:** microwave heating, innovation foods, nutritional macromolecules, flavor, safety risks

## Abstract

Microwave heating technology performs the characteristics of fast heating, high efficiency, green energy saving and easy control, which makes it deeply penetrate into the food industry and home cooking. It has the potential to alter the appearance and flavor of food, enhance nutrient absorption, and speed up the transformation of active components, which provides an opportunity for the development of innovation foods. However, the change of food driven by microwave heating are very complex, which often occurs beyond people's cognition and blocks the development of new food. It is thus necessary to explore the transformation mechanism and influence factors from the perspectives of microwave technology and food nutrient diversity. This manuscript focuses on the nutritional macromolecules in food, such as starch, lipid and protein, and systematically analyzes the change rule of structure, properties and function under microwave heating. Then, the flavor, health benefits, potential safety risks and bidirectional allergenicity associated with microwave heating are fully discussed. In addition, the development of new functional foods for health needs and future market based on microwave technology is also prospected. It aims to break the scientific fog of microwave technology and provide theoretical support for food science to understand the change law, control the change process and use the change results.

## Introduction

The microwave technology was first employed in the food business in the 1970s, and it is most widely used for thawing and drying foods. With the development of microwave technology, it began to be utilized in the puffing, sterilization and alteration of foods. The advent of microwave puffing method offers an opportunity to create an expanded snack product, which can change the food texture, and better preserve the nutrients and rich flavor. In 1976, Pillsbwry company of the United States introduced microwave popcorn for the first time, and then microwave foods such as potato chips, cakes, noodles, etc. appeared on the market in the mid to late 1990s. These foods have the characteristics of fast heating speed, low oil content, high product quality, uniform heating and environmental protection. Based on the above advantages, it is now very popular in daily life. However, different views believe that microwave heating will destroy the nutrients in the food and produce unknown substances, which also have potential health risks. For this purpose, this paper summarizes the effects of microwave on macromolecule nutrients (starch, lipid and protein) in food, in order to answer whether microwave heating has a negative effect on food nutrition, and to provide a comprehensive overview of the most recent research findings in order to provide a theoretical foundation for promoting microwave use in the food industry.

## Starch

Carbohydrate-rich foods and root vegetables, such as rice, wheat, corn, and yams, are examples of starchy foods. Microwave heating affects the vibration of groups in starch molecules through both thermal and non-thermal effects. The temperature effect affects the vibration intensity of polar groups in starch molecules, whereas the non-thermal effect primarily affects the vibration intensity of skeleton modes like the glucoside bond and pyran ring, as well as skeleton groups like C-O and C-O-H (1, 2). Microwave stimulates the development of free radicals at the C1 and C6 locations in starch molecules, as well as the structural modification of C1 free radicals, resulting in the production of more free radicals (3). Furthermore, the microwave sensitivity of different starch molecules varies, owing to differences in starch crystal structure and amylose concentration (4). Due to the heat resilience of amylopectin, which creates the waxy starch, waxy corn starch is less impacted by microwave heating (5). However, the features of microwaves that promote slow starch digestion are tightly related to microwave power and starch moisture: The fundamental reason for the creation of resistant starch is starch recombination generated by high-power microwaves, and the higher the water content in a given range, the higher the gelatinization degree and swelling power, and the higher the digestibility.

### Structure

**Figure 1:**
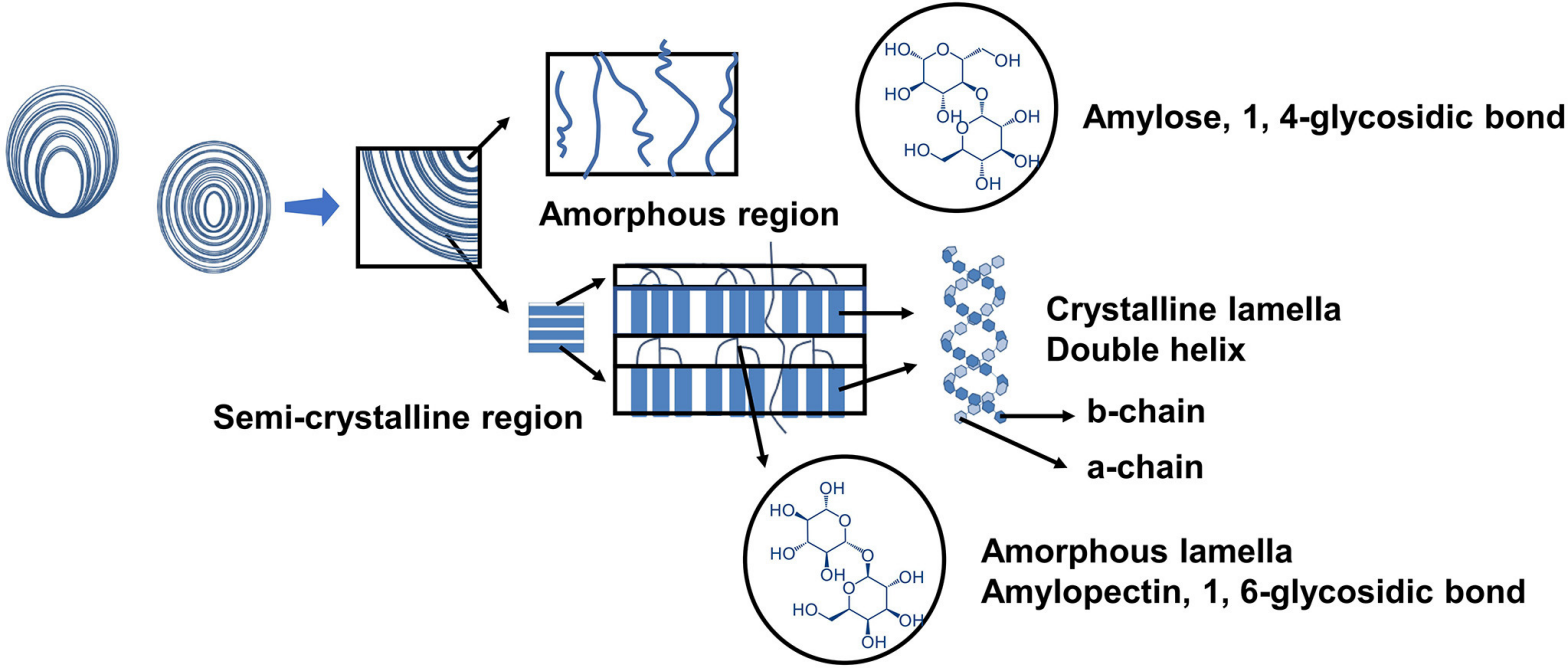
The structure of starch granule.

The starch system is a polycrystalline one. The crystallization area and amorphous area, the two main components of starch, alternate to form the semicrystalline area, which then alternates with the amorphous lamella to form starch particles. The structure of starch granules is shown in Figure 1. Microwave heating changes the polycrystalline structure of starch, mostly from ordered to disordered, and hence affects crystallinity, surface morphology, and other significant aspects. Microwave treatment, on the other hand, reduces the conversion of starch structure from ordered to disordered as compared to traditional heating. Amylopectin is the semi-crystalline portion of starch, and its molecular structure is the most important component in determining starch's physical qualities (6). Amylopectin degradation can be separated into two stages and is aided by microwave heating (7). The major degradation occurred in internal chain (amorphous region) at the first stage, the external chain (crystalline region) mostly destroyed at the second stage (6). The external chains (A chain) and short B chain of amylopectin are twirled into a double helix and exist in the crystalline domain, which affects the crystallinity. The inner chain is primarily seen in amorphous lamellae. The amorphous structure is primarily made up of amylose. Microwave treatment causes starch to transform from an ordered to a disordered structure, however the microwave's thermal and non-thermal actions have different impacts on starch structure. The rapid heating effect causes the double helix structure of amylopectin molecules to become more closely arranged in the crystalline layer, compressing the amorphous layer, whereas the non-thermal effect can protect the amorphous layer from the damage caused by rapid heating by causing irregular lamellar structure alternation (2). As a result of the combined action of microwave's fast heating effect and non-thermal effect, the influence of microwave on the ordered and disordered structure of starch is somewhere between conventional slow heating and fast heating. For example, following microwave heating, the content of amorphous structure of potato starch rose by 29% compared to the original starch, while the quantity of double helix structure reduced by 22%, both of which were between the fast and slow heating samples.

**Figure 2:**
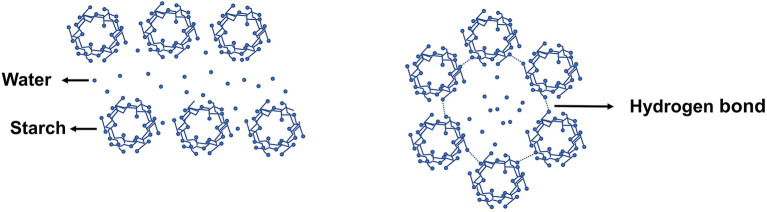
Crystal pattern of natural starch (type-A on the **left** and Type-B on the **right**).

**Figure 3:**
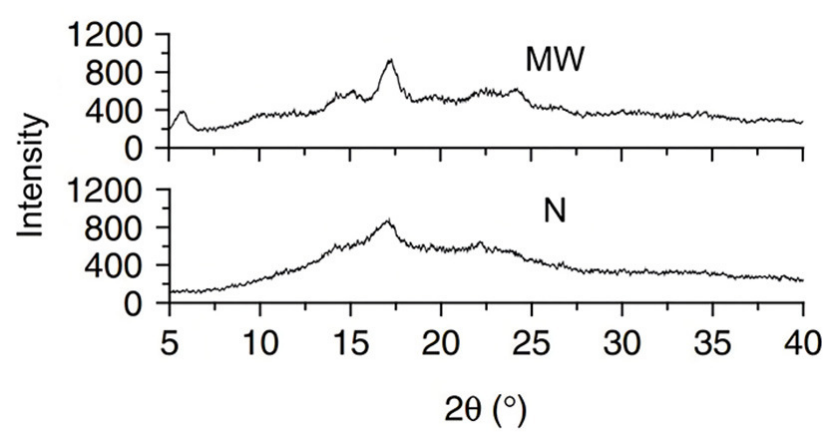
X-ray diffraction (XRD) of Indian horse chestnut starch granules. N, native; MW, microwaved (15).

The change in starch crystallinity crystallization type reflects the influence of microwave on starch crystal structure. In general, microwave heating lowers the crystallinity of starch, as shown in Table 1. After microwave heating, the relative crystallinity of white sorghum, maize, and other starches, for example, was seen to decrease (above 300 W). However, because of the thick structure, waxy corn starch crystallinity remained intact following microwave treatment (5). Natural starch crystallinity varies with plant kinds from various sources, however there are primarily four types (A, B, C and V-type). The majority of the change generated by microwave heating is from type-B to type-A, shown in Figure 2. Microwave heating, for example, transformed the crystallinity pattern of potato and Canna edulis Ker starch from type-B to type-A (13, 14), as shown in Figure 3. However, after using an optimized microwave-enzymatic hydrolysis process (800 W, 90 S), the crystal structure of potato starch changed from type-B to type-C (16). Because type-C is the intermediate state of continuous change from type-A to type-B, which can be transformed in some special methods or under predetermined conditions, and can also be regarded as a mixture of type-A and type-B, this change also conforms to the general change law of transformation from type-B to type-A.

**Table 1:** Effect of microwave treatment on starch crystallinity in food.

**Food species**	**Relative crystallinity (%) (natural starch)**	**Relative crystallinity (%) (microwave starch)**	**References**
Maize	19.58	2.91	(6)
White sorghum	25.88	18.08	(8)
Chinese chestnut	23.52	20.11	(9)
Wheat	36.81	27.53	(10)
Cassava	28.1	18.47	(11)
Potato	29.09	26.25	(12)

The existence of a significant number of intermolecular and intramolecular hydrogen bonds is strongly related to the structure stability of natural starch, and microwave heating can impact them primarily by modifying the water distribution and dynamic process in the granules (9, 17). The thermal stability of starch before gelatinization is determined by the quick heating action of microwaves, which can impede the breaking of hydrogen bonds between starch and water molecules, altering the macroscopic physical and chemical properties of starch (18). However, the vibrating motion of the polar molecules, on the other hand, promotes the breaking of hydrogen bonds, and the effect is generally stronger than the microwave's quick heating effect (19). Microwave's non-thermal effects on starch are limited to microcosmic features such molecule polarization, skeleton compactness, and water distribution in multilayer structures.

Because microwave treatment alters the structure and crystallinity of starch, it also alters the surface morphology and properties of the starch, such as viscosity, gelatinization, swelling force, oxidation resistance, and digestibility.

### Starch grain morphology

**Figure 4:**
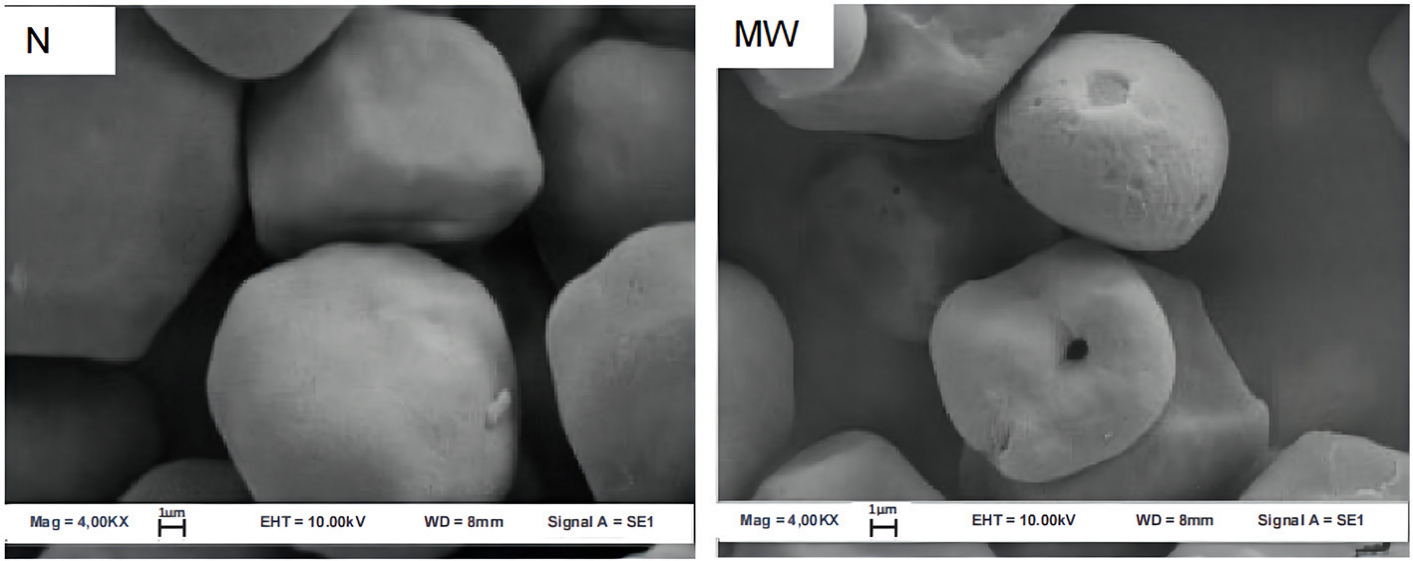
Scanning electron micrographs (SEM) of maize starch granules. N, native; MW, microwaved (20).

**Figure 5:**
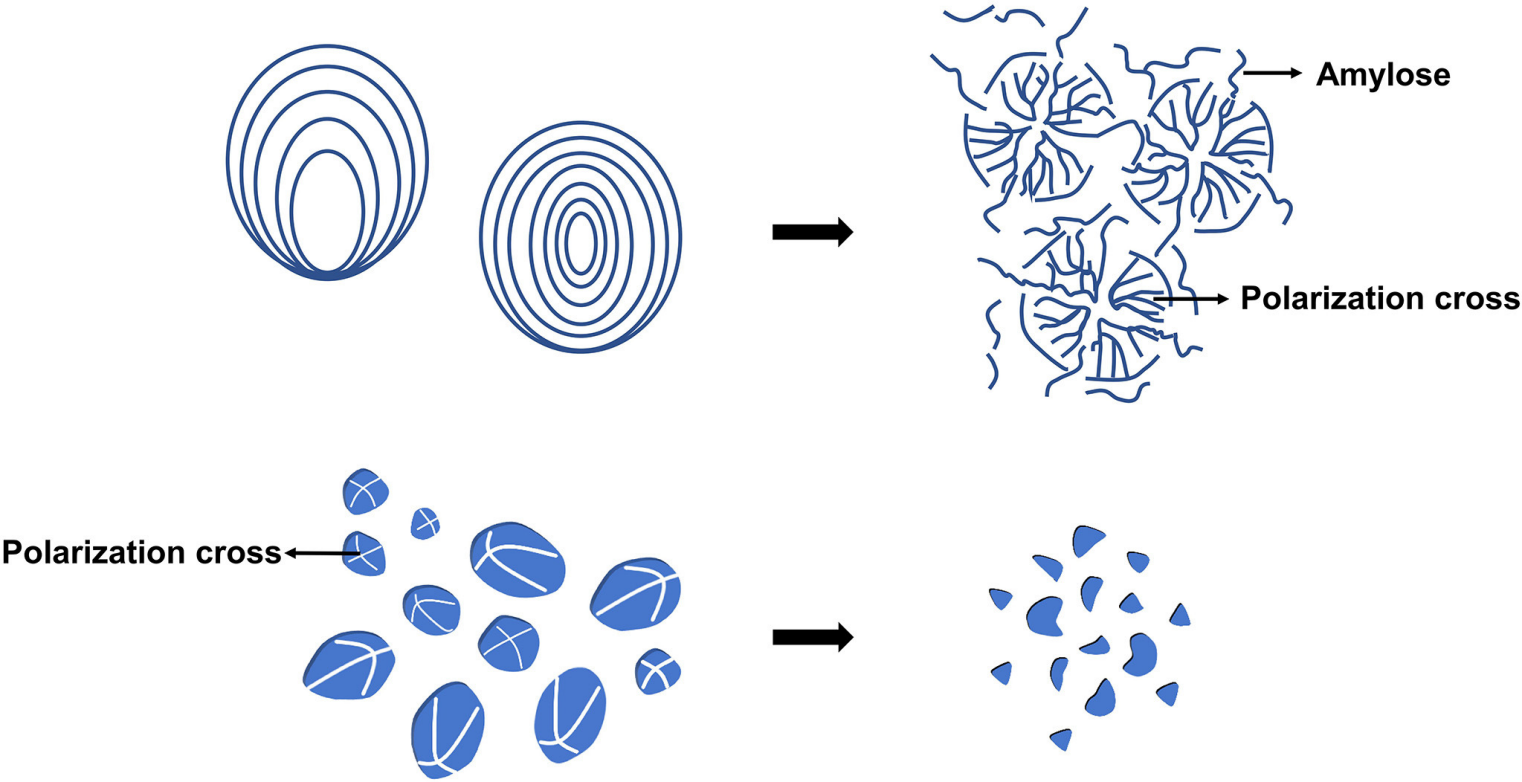
Morphological changes of starch grains treated by microwave.

Microwave treatment can degrade the integrity of starch particles, increasing the number of concave or folds on their surface, shown in Figures 4, 5. Because the weakest section of the particle breaks when it absorbs water and expands to a certain level, the internal starch polymer flows out of the fractured part and separates the particle shell, causing the surface to fold. For example, after microwave heating, there were many depressions or folds appeared on the starch grains surface of cassava and chestnut (9). At the same time, microwave heating causes the polarization cross characteristic of starch granules to vanish. The polarizing cross in the granules is caused by the radial arrangement of amylose and amylopectin. The difference in density and refractive index of crystal and amorphous structure reveals anisotropy when viewed with a microscope under polarized light, demonstrating birefringent phenomena. Because the vibrational motion of the polar water molecules breaks the lamellar arrangement during the microwave heating process, the particles' birefringent vanishes altogether (21, 22). The polarized cross of potato and white sorghum, for example, vanished following microwave treatment (23, 24).

### Properties

#### Viscosity

When heated, starch becomes more viscous. The hydrogen connections between the starch molecules in crystalline region and the starch molecules in amorphous region are broken when water molecules enter during heating. The starch-protein interaction vanishes, and the double helix straightens to generate a separation state, destroying the amylopectin crystal structure. This causes amylose with a tiny structure to exudate from the particles, resulting in increased viscosity and transparency. After microwave heating, for example, there were apparent aggregation and bonding behaviors between starch particles of chestnut and lotus seed (16).

However, the dissolved viscosity of microwave-processed starch food (dry, puffed) is frequently lower than that of conventional dry food. The starch viscosity with this treatment decreases after a period of increase, and the peak viscosity is lower than the final viscosity of untreated starch, as shown in Table 2. This is related to the degradation of starch particle structure and a decrease in relative crystallinity, which prevents starch from absorbing or binding water (16). Microwave may also encourage the creation of a double helix structure of the long chain segment of amylopectin or build a compound with other dietary components such as lipid and protein to prevent starch from expanding. At the same time, it was shown that as microwave treatment progresses, intermolecular and intramolecular hydrogen bonds in starch increase, implying enhanced starch association, reduced amylose exudation, and lower viscosity (26, 27).

**Table 2:** Effect of microwave treatment on starch viscosity in food.

**Food species**	**Viscosity (Pa·s) (natural starch)**	**Viscosity (Pa·s) (microwave starch)**	**References**
Cassava	1.9343	0.85433	(11)
Potato	8.2292	2.1250	(16)
Maize	302.7	200.2	(6)
Millet	785.3	448.6	(25)
Indian horse chestnut	3.540	1.689	(14)

Furthermore, the apparent viscosity of microwave-treated starch decreases with increasing shear rate and exhibits shear thinning behavior, which is linked to the progressive orientation of molecules in the flow direction and the breaking of hydrogen bonds formed in the amylopectin-amylopectin-water structure during shear (28, 29). After microwave treatment, the viscosity of starch is lowered, reducing the taste unpleasantness caused by high viscosity of food. Furthermore, the starch hardness is improved, making microwave method more suitable for the production of biscuits and other chewable foods that require a specific amount of hardness.

#### Expansion force

**Figure 6:**
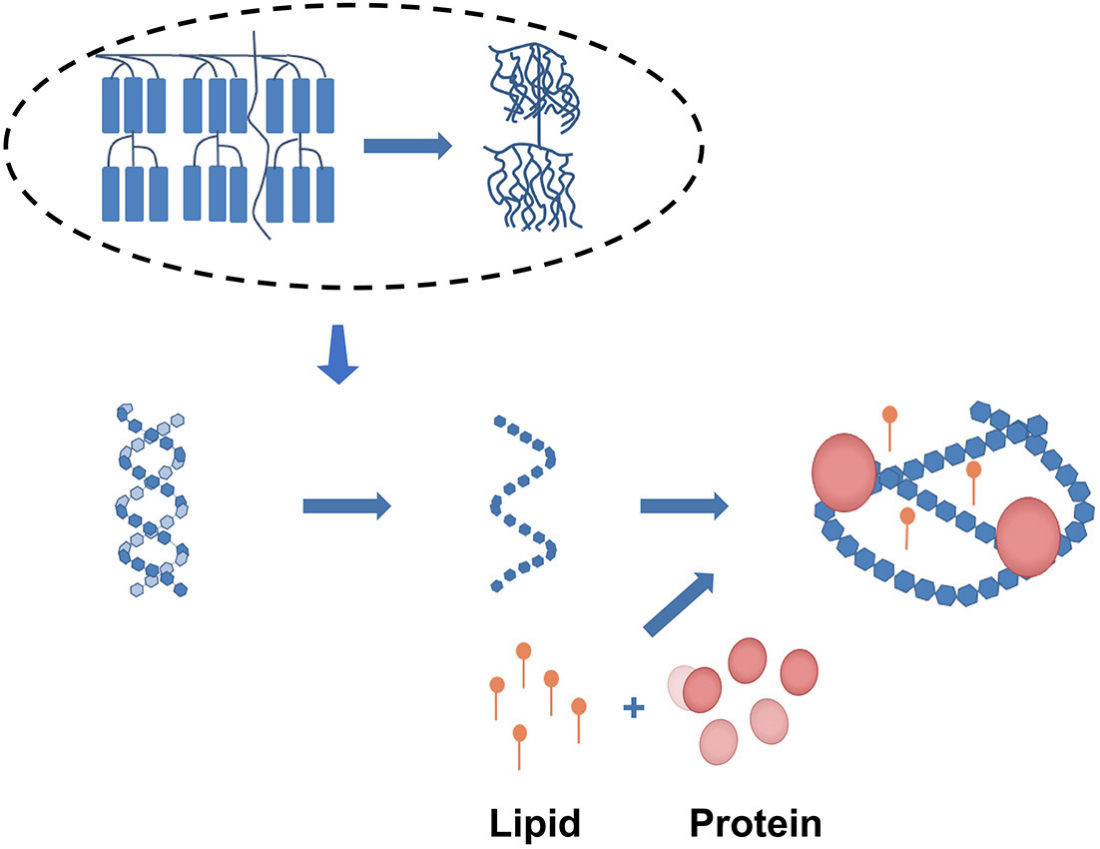
Starch forms a complex with proteins and lipids.

The interaction between the starch chains in amorphous domains and starch chains in crystalline region could be reflected by the value of swelling power, which depends on properties of amylose and amylopectin such as the molecular weight, relative content, branch length and degree of branching. Under conventional heating, the expansion force of starch will increase with the increase of heating temperature, but microwave heating can inhibit expansion by increasing contacts between amylose and amylopectin molecules, preventing water molecules from entering the inner region and reducing amylose dissolution (30). Because amylose works as a diluent, a high concentration of amylose or binding molecules will limit the expansion (31). At the same time, physical interactions between food's key components are common. These interactions may encourage the formation of V-type starch-lipid complexes or terpolymer starch-lipid-protein complexes with bigger molecular and weight structures from starch, lipids, and proteins, shown in Figure 6. These compounds can also prevent water molecules from entering the starch, reducing its swelling potential (32, 33). Aside from that, changes in other food elements throughout the microwave cooking process will have an indirect impact on expansibility. For example, in late microwave processing, protein forms a rigid gel network (34, 35). Furthermore, during microwave heating, the molecular vibration and rapid increase in temperature will cause the particles to rupture and form a polymer film covering the surface of the particles, preventing expansion. Simultaneously, because granule hydration does not keep up with granule expansion, the resulting stress induces granule rupture (26). When microwave heating was employed to treat wheat starch dispersions, for example, the grains fractured due to a lack of gelatinization expansion (22).

#### Gelatinization

When starch is heated, it absorbs water, swells, and disperses throughout the solution, forming an extremely thick paste known as starch gelatinization. Gelatinization is defined as the breakdown of hydrogen bonds, the loss of association, and the separation of the double helix when water is introduced into starch grains. The starch molecules are then distributed in water, resulting in a hydrophilic colloid solution.

The gelatinization mechanism and rheological properties of starch can be affected by microwave treatment. Two crucial parameters in the gelatinization process are gelatinization temperature and gelatinization enthalpy. The lowest temperature necessary for starch gelatinization is the gelatinization temperature. And, to some extent, the gelatinization enthalpy can reflect the energy needed to separate the double helices structures (13). Microwave gelatinization is often characterized by an increase in gelatinization temperature and a decrease in gelatinization enthalpy (ΔH). Corn, Chinese chestnut starch and other starches, for example, increased in gelatinization temperature while ΔH dropped under microwave treatment, as shown in Table 3. Microwave causes starch molecules to reorganize, resulting in tighter crystal regions, delaying the commencement of starch gelatinization. At the same time, as the microwave expansion force and amylose dissolution diminish, the starch gelatinization decreases. Microwave-treated lotus seed starch, for example, had a tight crystal zone structure and reduced amylose dissolution, altering the rheological properties of starch paste (38). Reduced starch gelatinization can improve food tensile strength and formability, as well as boost product crispness and strength. Microwave pizzas from the American Peel business and microwave pre-fried items from the Nichirei firm, such as Kelekai and Tianbuluo, for example, are both more crisp.

**Table 3:** Effect of microwave treatment on starch gelatinization parameters in food.

**Food species**	**Gelatinization temperature (°C)**	**ΔH(J/g)**	**References**
Maize	10.69↑	3.1↓	(6)
Chinese chestnut	2.79↑	2.49↓	(9)
Barley	12.7↑	2.8↓	(36)
Indian horse chestnut	20.18↑	1.4↓	(14)
Peanut	11↑	2.3↓	(37)
Millet	7.83↑	5.78↓	(25)

In addition, compared with the conventional heating treatment, which only leads to the gelatinization of the particle surface, microwave treatment will completely destroy them, because the microwave energy will affect the water molecules existing in the crystalline area of starch particles and enhance the cracking (39).

#### Oxidation resistance

Microwave-treated starch exhibits a greater DPPH free radical scavenging activity. One of the explanations is the lowering of free radical reactivity caused by the synthesis of new double bonds during starch breakdown. The phenolic compounds, on the other hand, may take precedence over the starch oxidation reaction. Microwave heat treatment can help to free bound phenolic chemicals in materials and increase phenolic exposure. The DPPH clearance of chestnut starch, for example, increased from 18.89 to 29.02 percent (14). The increased antioxidant activity of starch serves to lower the degree of oxidation of lipids and other dietary components, preventing peroxide damage to the body.

#### Digestibility

Heat treatment improves the digestibility of starch in general. Because heating leads to wide cracks and deep cavities on the granule surface, which facilitates access for the starch hydrolyzing enzymes to starch chains and accelerates the digester process (25). However microwave treatment can diminish the digestibility of starch, causing it to have slow digesting properties, as seen in rice and lotus seeds, which compared with conventional heating, have both increased their resistant starch (RS) and slow digestibility starch (SDS) (40).

The process by which the microwave slows the pace of starch digestion can be explained in two ways. On the one hand, after microwave heating, amylopectin degrades to create additional amylose and forms an amylopectin-polyphenol complex with polyphenols in the system, resulting in a high-amylose, heat-resistant, and slow-digesting product. To lotus seed, for example, could produce a heat-stable slowly digestible high-amylose maize starch by adding certain amount of tea polyphenols (26). At the same time, an increase in amylose molecular weight can cause glucan chains to recombine and form an organized semi-crystalline region structure, slowing digestion. High-power microwave treatment, compared to typical cooking storage, enhances the molecular sequence and cyclical amorphous crystal structure, according to studies. This alteration encourages the creation of amyloid chain domains with a medium density accumulation and delayed digestion (41). In conclusion, the sluggish digestion of starch is caused by the breakdown of amylopectin and the creation of high amylose. After microwave treatment, the amylose and RS contents of potatoes increased to 35.06 and 27.09%, respectively (42). These are also the reasons why waxy and low-amylose rice's starch hydrolysis in life is often faster and more complete than intermediate- and high-amylose rice's.

Microwave-induced reductions in starch digestibility, on the other hand, can be related to changes in starch's multistage structure during digestion. Not only does digestion hydrolyze the starch matrix, resulting in porous substrates and a reduction in polycrystalline and nanoscale order, but it also reorganizes the starch chain, resulting in a crystalline transition from untreated starch type-A to type-B and the formation of new molecular structures. As a result, matrix hydrolysis and molecular recombination, which occur simultaneously during the process, play a major role in starch digestion. Unlike normal starch, the microwave processed starch's polycrystalline component is preferentially digested (43). However, when compared to starch treated with ordinary heating, microwave-treated starch has a higher molecular recombination ability during digestion, resulting in slower digestion. Conversion from type-B to type-A+B (type-C) can also enhance the concentration of RS while decreasing digestibility by increasing crystallization area and resistance to enzymatic hydrolysis (27, 44).

After microwave heating, the content of resistant and slow digestible starch increases, and it is not enzymatically hydrolyzed in the small intestine, but it can be fermented with volatile fatty acids. As a result, it can lower the body's glycemic index and weight, making it ideal for diabetics and beauty enthusiasts.

It is not difficult to find that the particle shape, crystallinity, rheological behavior, gelatinization temperature, enthalpy and digestibility of starch all depend on the starch type and its moisture content, microwave treatment time, treatment temperature and absorbed microwave energy. Therefore, future research should systematically focus on the physical, chemical and structural changes of different kinds of starch under microwave treatment under different parameters, so as to better understand the specific changes related to the parameters of starch during microwave heating, which will be more helpful to predict the overall behavior of starch on this microwave processing, and help design and improve the processing of starch and starch products and the quality of final products.

## Lipids

Daily high fat food is mainly the food with high oil content and fried, including peanut, fatty meat, animal viscera, butter products and so on. Because lipids have a low specific heat and heat up quickly, they are particularly vulnerable to microwave heating. Microwave heating, on the other hand, has a lower impact on oil than traditional heating. The polar molecules content in the oil is lower, but it is the main heating under microwave, because microwave heating can convert mechanical energy generated by asymmetric vibrations of polar molecules into heat energy. To minimize lipid oxidation, microwave energy can also inactivate lipoxygenase and eliminate hydrogen peroxide. Microwave treatment, for example, lowered the oxidation rate of rice bran oil when compared to traditional heating (45).

### Structure

Microwave heating will trigger lipids oxidation, leading to lipid polymerization and thermal oxidative decomposition. However, compared with conventional heating, microwave has a lower degree of lipid oxidation, because on the one hand, heating will accelerate oxidation, and on the other hand, microwave can enhance the antioxidant capacity of lipids and delay oxidation. Microwave-treated vegetable oils, in general, produce hydrogen peroxide and secondary oxidation products quickly. Because lipid secondary metabolites can harm the body, lipid oxidation should be prevented as much as possible during food processing. Furthermore, lipolysis, which might result in a rise in acid, is a significant alteration in lipid during microwave heating. Lipolysis, lipid polymerization, and heat oxidative breakdown will all have an impact on lipid composition and characteristics.

### Lipid composition

**Figure 7:**
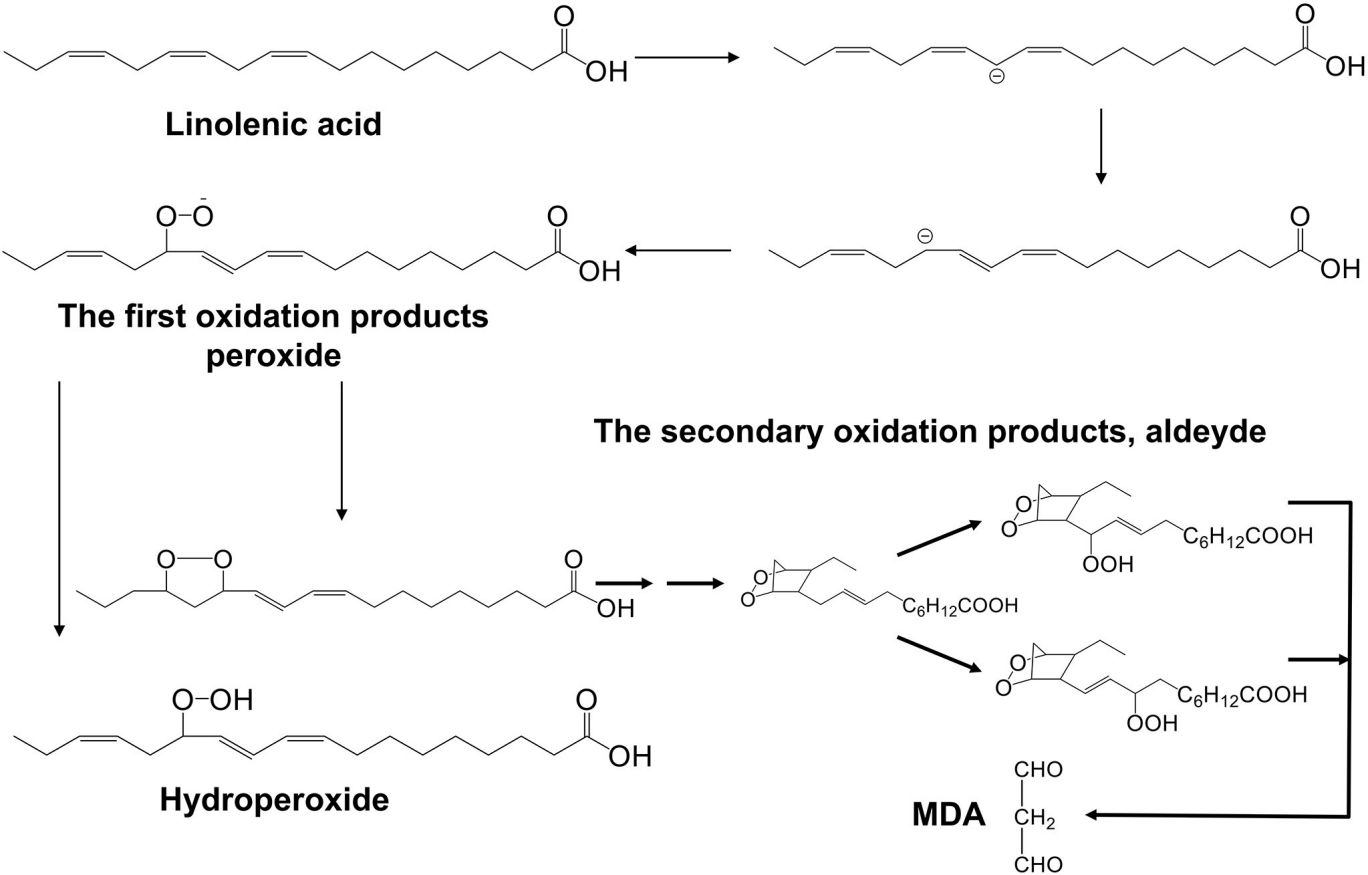
Oxidation process of polyunsaturated fatty acids (taking linolenic acid as an example).

Fats and lipoids are two types of lipids. Because lipolysis and lipid oxidation are common, the total content of lipids, as well as the contents of fats and lipoids, are all reduced following microwave treatment, while the quantity of fatty acids is increased and the composition of fatty acids changes, the oxidation process of polyunsaturated fatty acids is shown in Figure 7. The content of crude fat and phospholipid in trichosanthis seed, for example, dropped after microwave treatment, whereas the content of free fatty acids increased (46).

Temperature, oxygen, the unsaturated fatty acid ratio, and other factors influence lipids loss during microwave therapy. Because of the high temperatures and copious oxygen involved in heating, baked foods lose substantially more phospholipids than microwaved foods. Phosphatidylcholine (PC), phosphatidylethanolamine (PE), phosphatidic acid (PA), and sphingomyelin (SM), which are abundant in polyunsaturated fatty acids, were more affected by microwave than other components in black egg yolk (47). In addition, other factors, such as water evaporation from the treatment process, also affect lipids content, because lipids oozes out when water evaporates. For example, the lipids loss of hot air drying fish was more serious than that of microwave drying fish (48).

Microwave treatment of free fatty acids in food can increase their content and change their composition. Monounsaturated fatty acids (MUFA) and polyunsaturated fatty acids (PUFA) proportions decrease, whereas saturated fatty acids (SFA) and trans fatty acids (TFA) proportions increase. The rapid oxidation of lipids and the loss of water in the microwave process, which fatty acids spread and exchange between fat and water, causes this occurrence. The retention rate of unsaturated fatty acids is higher when compared to standard cooking and drying methods such as baking and air drying (49). Buckwheat and other foods arfter microwave, for example, had a larger PUFA proportion than that after conventional heating, although they had lower total lipids content, SFAs, MUFAs, and PUFAs under microwave, as shown in Table 4 (51).

**Table 4:** Effect of microwave treatment on lipid composition in food.

**Food species**	**Conventional heating**		**Microwave heating**		**References**
	**MUNA/SFA**	**PUFA/SFA**	**MUNA/SFA**	**PUFA/SFA**	
Hamburgers	0.023↓	0.01↓	0.381↑	0.009↓	(50)
Buckwheat	0.05↓	0.025↓	0.03↓	0.024↓	(51)
Grass carp	0.172↓	0.302↑	0.114↓	0.353↑	(52)

Furthermore, the content of unsaturated fatty acids influences microwave heating rate, with the larger the content, the faster the heating rate. Sunflower oil, for example, which has a larger proportion of unsaturated fatty acids, heats up faster, whereas peanut oil, which has a lesser content, heats up more slowly (53).

### Acid value and peroxide value

The acid value and peroxide value of lipids in microwave foods have a significant impact on food safety. The more fat is oxidized, the more chemicals like aldehydes, ketones, and acids are generated, causing more cell damage. Cells can be damaged by partial oxidation products of lipids, SFA, hydroperoxides, and malondialdehyde, however, MUFA and PUFA can protect cells (54).

The content of fatty acid grows as lipids decompose, and the acid value of the oil increases as the microwave intensity and time increase, but the pace of growth is sluggish. On the other hand, the peroxide value follows a zigzag pattern of increasing-decreasing-increasing. Because of their instability, peroxide, the main result of lipid oxidation, is transitory. When lipids are heated in the microwave, they oxidize and breakdown to create peroxide, which is quickly degraded into oxidized secondary products as the temperature rises. The peroxide value tends to rise when the rate of peroxide generation exceeds the rate of peroxide decomposition; otherwise, it tends to fall. Furthermore, as the heating temperature and duration increase, the acid value of fatty foods increases, and the degree of influence is related to the unsaturated degree of oil. For example, following microwave heating, the acid value of tea seed oil increased and was related to temperature and heating duration. Meanwhile, the peroxide value rose first, then fell, and the larger the microwave power, the shorter the time it took to reach its maximum value (55).

Because of the quick disintegration of the primary product hydroperoxide, various subsequent products can represent the oxidation degree of lipids in addition to acid and peroxide values. Microwave heating usually further degrades lipids *via* fission, dehydration, and the generation of free radicals, resulting in hydrocarbons, ketones, and aldehydes, among other things. Malondialdehyde is a common secondary product of lipid oxidation, and its synthesis is influenced by the temperature, power, and duration of microwave heating. The concentration of malondialdehyde increases initially and subsequently drops during microwave heating, which is related to its volatility (56).

Although the acid value and peroxide value of dietary lipids will invariably rise as a result of microwave processing, but compared with conventional heating, the acid value and peroxide value are lower, and we can reduce the degree of lipid oxidation by controlling temperature, power, time, water and other factors. Food treated with a short time microwave, for example, had much lower carbonyl value and trans-fatty acid level than food treated with a long time microwave (57).

### Lipid oxidation

Microwave heating boosts lipid antioxidant capability and lowers lipid peroxidation. The following are the three main mechanisms: Microwave heating, for starters, can produce antioxidant active molecules to take part in the reaction of free radicals prior to the lipids. The other option is to lower the amount of reaction catalyst needed by improving metal chelating capacity. Third, by decreasing the action of oxidase, it can prevent the enzymatic oxidation of lipids.

To begin with, microwave heating promotes the formation of antioxidant active components in oil, such as carotenoids, phenolic compounds, and other chemicals. This is because microwave heating causes intense movement of polar molecules, which breaks the cellular structure of the food matrix and allows digestion enzymes to enter. The extraction rate then increases as the biological accessibility of these components improves (58). Microwaves also cause the protein's phenolic binding sites to be disrupted, allowing additional phenol to be released. These active chemicals take part in the reaction of free radicals before they react with lipids, removing free radicals and preventing lipid oxidation (59). At the same time, vitamin E has been associated to free radical disruption events and the formation of antioxidant dimers, and the retention rate of water-soluble and thermally unstable vitamins, such as vitamin E, is higher after microwave cooking than after conventional heating (60, 61). For example, after microwave processing, the total phenol content of trichosanthes seed oil increased, resulting in improved antioxidant capacity (46). Although the phenolic compounds of rice bran treated by microwave decreased slightly, the antioxidant capacity was still increased due to the enhanced extraction effect of carotenoid and chlorophyll (45).

Microwaves also increase lipid oxidation indirectly by changing the protein characteristics of foods. It can, for example, accelerate the Maillard reaction, boost proteins' metal chelating ability, and inhibit the activity of lipid oxidase. The lipoxygenase activity of trichosanthis seed oil, for example, was reduced by 90.30% after microwave heating (46). Apart from that, a minor amount of amino acid derivatives was discovered in the fat of the bran after microwave cooking, which could be Maillard reaction products and could be used as antioxidants to preserve oil from oxidation (45).

Microwave pretreatment and crushing is currently employed in the oil processing sector because it not only improves oil extraction rates, but also boosts oil oxidation resistance, reducing the impact of oil oxidation on flavor and safety.

## Protein

Protein is the building block of life and one of the most important nutrients for the human body. Microwave heating, in contrast to traditional heating, uses a combination of thermal and non-thermal effects to alter complicated protein structures by disrupting intramolecular interactions. These alterations will then have an impact on the characteristics of proteins.

### Structure

**Figure 8:**
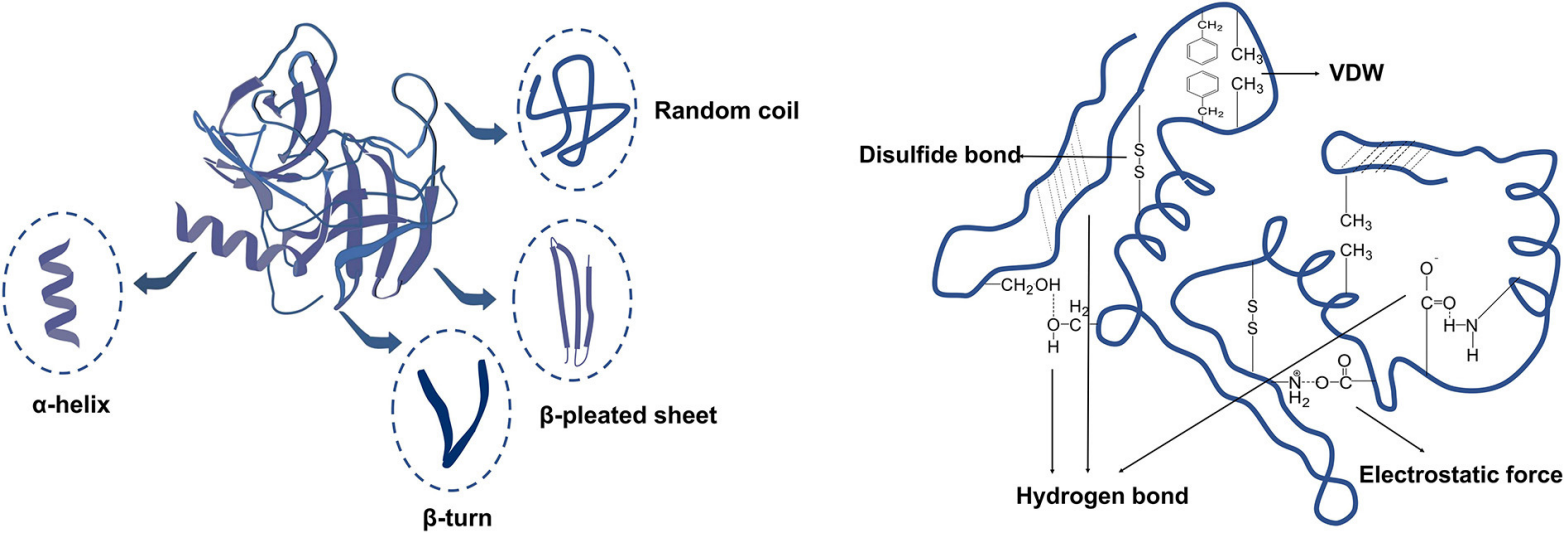
Secondary structure and intramolecular forces of proteins.

By creating free radicals and larger or smaller molecules during microwave heating, electric and electromagnetic fields can cause conformational changes in proteins, damaging the primary, secondary, tertiary, and quaternary structures of proteins, the secondary structure and intramolecular forces of proteins is shown in Figure 8. Compared with conventional heating, microwave can accelerate the unfolding of proteins. Under high-powered microwave heating, the protein disulfide link breaks, exposing the hydrophobic core residue to the solvent, and the protein depolymerizes. The percentage of ordered and disordered structure will shift during this process, primarily from ordered to disordered. Because the hydrogen connection between carbonyl (C=O) and amino (-NH2) contributes to the stability, the α-helix has a regular ordered structure, and the more of it there is, the more stable the protein's secondary structure is (62, 63). The following are the structural alterations in proteins caused by microwave treatment: Random coil rises, α-helix decreases, β-sheet and β-turns increase first and subsequently decrease. In other word, the α-helix changes to β-sheet and β-turns throughout this procedure, while the β-chain changes to random coil (64). A possible mechanism is proposed: the various effects of microwave synergistically weaken the previously intra-molecular and inter-molecular forces including hydrogen bonding, disulfide bonding, and hydrophobic interactions, which led to the formation a new structure by the rearrangement of the molecular forces (65). For example, under microwave heating treatment of lotus seed and pigeon bean flour, the ordered structure of protein decreases and the disordered structure increases, as shown in Table 5.

**Table 5:** Effect of microwave treatment on protein structure in food.

**Species**	**α-helix (%)**	**β-sheet (%)**	**β-turn (%)**	**Random coils (%)**	**References**
Lotus seed	20.59↓	7.36↓	9.46↑	18.49↑	(66)
Myofibril	22.5↓	9↑	4.2↑	4.9↑	(1)
Rice	0.5↑	3.5↓	0.2↓	1.6↑	(67)
Barley grains	0.003↓	0.014↓			(68)
Fiber	8.71↑	5.8↓	11.58↓	10.55↑	(69)
Peanut flour	2.7↓	1.4↑		4.3↑	(70)
Soybean	4.8↑	9.49↓	3.8↑	0.89↑	(71)
Quinoa	1.5↓	0.7↑	0.3↓	1.3↑	(72)
Pigeon pea	3↓	5↓	2↑	5↑	(73)

However, during the treatment procedure, microwave may cause glucan glycosylation, which is aided by protein. The effect of the microwave on the secondary structure of the protein will be reduced, and only the tertiary structure will be affected. Because high quantities of dextran crowd the system and produce steric hindrance, which prevents excessive denaturation. For example, after the Maillard reaction was triggered by microwave, no substantial modifications in the secondary structure of rice protein were identified (67). Furthermore, the Maillard process was discovered to transform the protein structure from disorder to order to some extent. In the microwave-induced Maillard reaction of rice residue protein, for example, the α-helix increased while the β-sheet and β-turns decreased (74).

**Figure 9:**
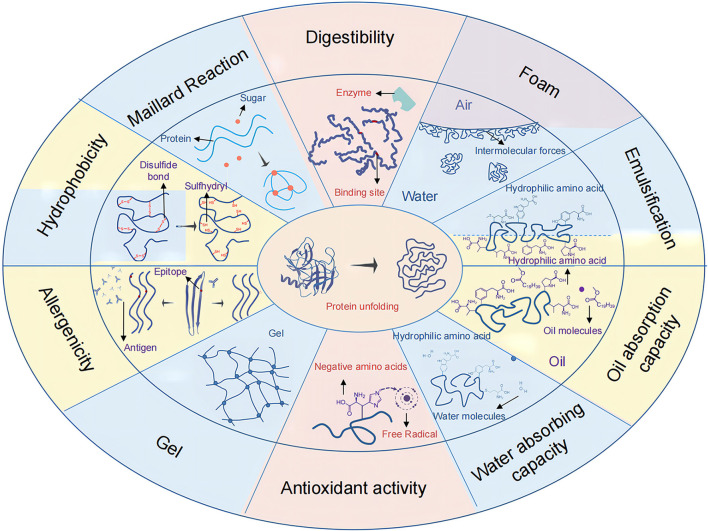
Effect of microwave heating on protein structure and properties.

Microwave treatment can alter the structure of proteins, which can affect properties such as hydrophobicity, digestibility, emulsification, foaming, gel resistance, oxidation, and allergenicity. Also affected is the Maillard process between protein and decreasing sugar, shown in Figure 9. For example, a high β-sheet and α-helix ratio causes digestive enzymes in the gastrointestinal tract to be less accessible, resulting in poorer protein value and utilization.

### Properties

#### Hydrophobicity

Protein hydrophobicity rises when heated with high-powered microwaves. At the same time, compared with conventional heating, a higher degree of hydrophobicity can be obtained (75). This is because when polar protein molecules collide, free radicals are produced, causing disulfide bonds to dissolve and sulfhydryl groups to form. Protein hydrophobicity increases as the total sulfhydryl group content increases (76). The majority of hydrophobic residues are found in the interior of protein's structural structure (4, 77). As the structure unfolds during microwave treatment, more non-polar amino acids are exposed on the surface, increasing the hydrophobicity (76). At the same time, the repulsion of more homogeneous charges on the protein surface will limit molecule aggregation, reduce particle size, and increase the stability of the solution due to the increased Zeta potential of microwave protein solution. The degree of enrollment of hydrophobic groups decreases at this period, but surface hydrophobicity rises (78). The protein water solubility of soybean meal, for example, reduced from 94.4 to 48.1% following microwave treatment, whereas the disulfide bond content increased by 42% (79).

However, after a period of microwave heating, protein hydrophobicity decreases rather than increases. On the one hand, this is because certain sulfhydryl can be converted to disulfide bonds with the help of active free radicals, resulting in a reduction in overall sulfhydryl group content and hydrophobicity. In the presence of oxygen, the sulfur group becomes more reactive, and the distance between the groups shrinks, making disulfide bonds easier to form (80). The hydrophobic residues, on the other hand, are prone to aggregation when microwaved at high power or over long periods of time (81). For example, as microwave power and time were increased, the surface hydrophobicity of beef proteins increased at first, then reduced slightly (13).

After microwave heating, the hydrophobicity of protein increases and the interaction with low polar solvents becomes stronger, so it is more suitable for cake ingredients, salad seasonings and other oil-based ingredients. Protein hydrophobicity also has an impact on its antioxidant, foaming, gelation, and oil absorption abilities, and these properties are linked to the appearance and flavor of food. As a result, in the food processing process, careful attention should be paid to the time and power of microwave treatment to avoid excessive protein denaturation and aggregation caused by excessive power and heating time, excessive protein denaturation and aggregation will alter the hydrophobic properties and cause the final product's appearance and flavor to differ from expectations.

#### Digestibility

The digestibility of protein can be improved by microwave treatment. One of the most important reasons is that microwaves can alter enzyme function, resulting in increased protein-protease interaction. Microwaves alter enzyme activity in four main ways. (1) Microwave treatment inhibits thermally unstable enzymes due to heat denaturation (82). (2) Due to molecular rearrangement and protein unfolding, microwave may render specific protein locations more vulnerable to enzymatic hydrolysis. (3) The microwave-treated protein sample's reduced particle size can provide greater surface area and expose more cleavage sites for digesting protease activity. (4) After microwave treatment, a protein with a high Zeta potential developed, which helped to stabilize the protein suspension and prevent protein aggregation in water, increasing the number of exposed particular sites and the likelihood of protein-protease interaction (73). For example, after microwave heating, the protein of lotus seed unfolded, exposing more cleavage sites and increasing the degree of hydrolysis (6).

Protein unfolding and exposure to the enzyme reaction can also be caused by conventional heating. However, conventional hydrolysis takes longer to achieve the same degree of hydrolysis as microwave. This is because microwave non-thermal coupling can promote the breaking of non-covalent bonds in protein molecules, speeding up protein unfolding. Protein hydrolysis is best above 70°C, according to some studies, but protease is inactivated at this temperature, so it must be kept below 65°C. The final enzymatic hydrolysis result demonstrates that microwave heating with both thermal and non-thermal effects is clearly better to traditional heating using a conduction heat source (83).

However, as the microwave temperature, time, and power are increased, the digestibility of the protein decreases, because the increased heat treatment leads in complete denaturation of proteins. Then, through hydrophobic and electrostatic interactions, cross-linking reactions take place between proteins, transforming them into larger molecular weight aggregates, an insoluble three-dimensional network. Protein endonuclease will be more difficult to access as a result (84). This phenomenon is less likely to occur in microwave than in traditional heating because microwave's non-thermal effects contribute to appropriate denaturation of protein with modest aggregate extent. In general, non-thermal coupling can encourage protein molecules to break their non-covalent interactions, whereas a significant thermal influence can cause protein refolding or aggregation (4). Furthermore, the Maillard reaction has an inhibitory effect on proteolytic enzymes and can reduce digestibility through a mechanism similar to cross-linking aggregation between proteins (85). For example, milk protein began to aggregate after an 8 min microwave treatment, and the degree of hydrolysis and digestibility both decreased (86).

#### Antioxidant activity

Microwave treatment can enhance the antioxidant ability of protein, which is related to the fact that microwave can promote protein hydrolysis to produce more active peptides and enhance the metal chelating ability of protein. At present, it has been confirmed by experiments that compared with the unprocessed protein, the total antioxidant capacity (TAC) of hydrolysates of fish protein and shrimp protein after microwave treatment showed an increasing trend (87, 88). At the same time, compared with conventional heating, microwave protein has a higher DPPH value (75).

After protein hydrolysis, active peptides having antioxidant activity are generated. They can combine with free radicals to generate more stable products, as well as provide an extra source of protons and electrons for oxidation processes to keep the REDOX potential high (89). Microwave speeds up the hydrolysis of proteins into peptides, allowing more reactive species and electron-dense peptide bonds to reach the functional side of the chain (90). Microwave treatment can also cleave peptides into smaller molecular weight peptides, which have stronger antioxidant properties and are easier to permeate the intestinal barrier to perform biological functions (91).

The electrons, hydrogen-bonding characteristics, and position of the amino acids, as well as the steric properties of the amino acid residues at the C- and N-termini, all affect the antioxidant activity of peptides. Hydrophobic amino acids (Leu, Val, and Phe), hydrophilic and basic amino acids (His, Pro, and Lys), and aromatic amino acids (Phe and Tyr) all contribute to the improvement of antioxidant activity in the peptide sequence (92). Microwaves can increase the antioxidant activity of peptides by exposing hydrophobic residues and some polar charged amino acids to the peptide's terminal.

Furthermore, the ability of proteins to chelate metals has an impact on their antioxidant activity. Metal ion chelating (MIC) activity is attributed to peptides containing sulfhydryl amino acids, which can bind heavy metals and diminish their pro-oxidant action. Protein rearranges and releases the encrypted Sulfur peptide to grab metal ions during microwave treatment, resulting in improved MIC capacity (88, 93). The Salvia hispanica protein, for example, have higher MIC activity than conventional heating (75). However, several investigations indicated that when the protein was microwaved again during the enzymatic hydrolysis step, no increase in metal chelation was detected. This is because when microwave time, temperature, and power increase, protein reaggregation occurs and enzymatic proteolysis is prevented, resulting in a decrease in active peptide release (57, 94). Finally, some of the products of the Maillard process generated by microwaves have antioxidant characteristics.

Microwave-induced alterations in protein antioxidant capacity have an indirect effect on lipid oxidation, as some proteins can act as antioxidants after being microwaved. Metal chelation, for example, can help non-absorbed proteins contribute to the free radical scavenging mechanism of lipid antioxidant activity. Microwave protein's antioxidant properties can significantly reduce nutrient loss caused by oxidation of other substances in meals, as well as prevent damage caused by peroxidation.

#### Maillard reaction

Microwave treatment can increase the occurrence of food Maillard reaction (89). Because the active sites of Maillard reaction are mostly located in the internal regions of protein structures, and the extension microwave heating time exposes these sites. At the same time, under the high-power microwave treatment, the protein expands, which increases the probability of effective collision between it and sugar molecules, enhancing the Maillard reaction (95). For example, the activation energy of RP-dextran Maillard reaction of rice protein treated by microwave was lower than that of conventional heating (67). The Maillard reaction products can improve flavor, some can increase the antioxidant capacity and reduce the damage caused by peroxide to the body, but there are also some reaction products that are carcinogenic and harmful to the body's health. Moreover, the Maillard reaction process is complicated and there are many influencing factors. Therefore, at present, microwave-induced Maillard reaction still has uncontrollability and uncertainty of product properties. It is vital to undertake in-depth research on the microwave-induced Maillard reaction's reaction process and products if we wish to employ it to improve particular food features. At the same time, some of the negative effects of the Maillard reaction make it important to prevent this type of reaction from occurring in the processing process, which limits the use of microwave heating technology. However, we may explore decreasing the heating time, lowering the power, or adjusting some internal food parameters (starting moisture content and pH value), as well as adding some compounds (mercaptan compounds) to the pretreatment materials to reduce the incidence of Maillard reaction.

#### Allergenicity

**Figure 10:**
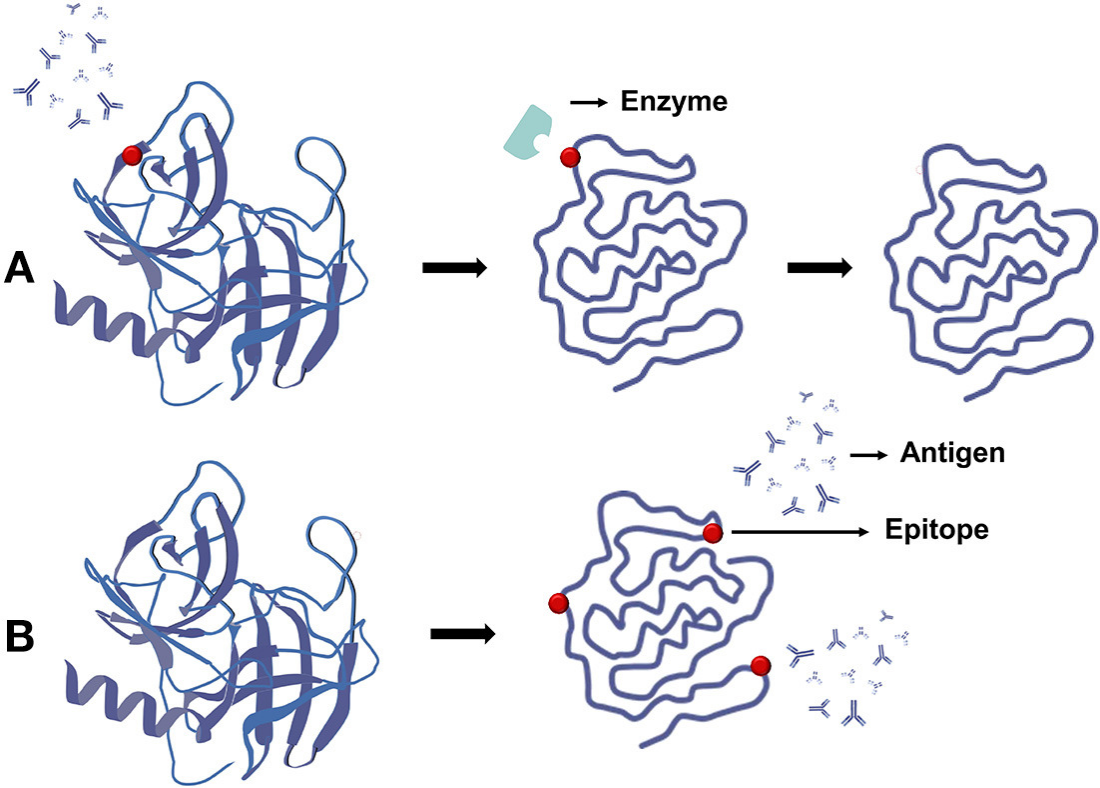
Effect of microwave on protein allergenicity. **(A)** Microwave promotes the degradation of protein epitopes and reduces allergenicity. **(B)** Microwave promotes the generation of new antigenic determinants and increases allergenicity.

Antigenicity of proteins is induced by the presence of epitopes, which are particular sequences in allergen proteins that, when recognized by the immune system, elicit allergic reactions. One reason microwave treatment reduces allergenicity is that it causes protein aggregation and structural changes, which prevent epitopes from being targeted. The other is that microwaves can reduce natural protein immune responses by destroying particular allergen epitopes through enzymatic hydrolysis. Microwave treatment can disrupt disulfide links in proteins, lowering their stability and making allergens more susceptible to enzyme breakdown, shown in Figure 10A. Although the raw protein has several protease digestive sites, the aggregated structure makes potential cleavage sites unavailable until the protein is denatured. Microwave decreases allergenicity by increasing the accessibility of sequence epitope proteases (87). For example, after microwave treatment, the antigenicity of gliadin, a proline protein linked to celiac disease in patients with gluten intolerance, was dramatically reduced (96). The hypersensitivity of prawn myosin also disappeared after microwave treatment (87). However, after microwave heating (500 W, 1–3 min), the allergenicity of tree nut proteins such as almond, cashew, and walnut remained unchanged (97). The reason for this is that the corresponding epitopes in allergenic proteins were not responsive to microwave heating, even when heated for a long time or at a high temperature.

Microwaves, on the other hand, may increase allergenicity. Because the unpredictability of the protein structure is unfolding, new binding sites may open up. Microwaves, in other words, induce denaturation of natural allergens, which causes proteins to develop new epitopes or make previously hidden epitopes available (crypt antigens), shown in Figure 10B. Microwave-treated peanuts and milk, for example, were shown to be more allergic in one study (98, 99).

The key aspect determining the bidirectional regulation of protein allergenicity by microwave is the type of meal. However, because there have been few studies on the subject, it is impossible to say which foods are less allergic and which are more allergenic after being microwaved. To avoid the occurrence of user allergy, special attention should be paid to the change in food allergenicity when using the microwave to process food. Furthermore, foods whose allergenicity has been shown to increase after being microwaved should be avoided being microwaved.

#### Water absorption and oil absorption capacity

Protein's propensity to absorb water and oil impacts not only the taste and flavor of food, but also the characteristics of other elements in food, such as the degree of gelatinization of starch. Microwave heating, according to studies, alters a protein's ability to absorb water and oil, owing to a change in protein structure. Protein depolymerization may expose more polar and non-polar amino acids, boosting the protein's interaction with water or oil molecules and so promoting water and oil absorption.

Water absorption capacity (WAC) is related to the presence of polar amino acids at primary cites of the protein-water interface. The uncoiling of hydrophilic domains of protein and the exposure of more polar amino acids after microwave treatment can lead to an increase in water absorption. Because of the accelerated expansion of protein, microwave protein can obtain higher water absorption capacity than conventional heating (100). For example, after microwave treatment, the WAC of red bean, potato etc. increased, as shown in Table 6. Because of the varying hydrophilic structures, the effect of microwave on the WAC of different proteins varies. Furthermore, once a certain level of water absorption capacity has been achieved, it is no longer increased. The ingested water will be released as the protein denatures and unfolds, and then promptly absorbed by carbohydrates and fiber from grains and legumes. The degree of gelatinization of starch will be affected by this moisture, which dictates the final texture and size of baked goods.

**Table 6:** Effect of microwave treatment on water absorption and oil absorption capacity of protein in food.

**Species**	**WAC (ml)**	**OAC (ml)**	**References**
Rice bran	0.2↓	0.1↑	(100)
Red bean flour	0.889↑	0.308↑	(101)
Wheat flour	1.173↓	0.419↑	(101)
Haleem wheat flour	0.139↑	0.02↓	(101)
Indian horse chestnut	3.88↑		(14)
Potato	3.62↑	0.078↓	(12)
Lotus seed	69.8↑		(4)

Oil absorptivity (OBC) is an important functional characteristic as it is essential to improve mouth feel and flavor retention. Microwave heating can increase the strength of protein binding oil molecules because more non-polar side chains are exposed during this process, increasing oil absorption by binding the hydro-carbon chains of lipids (100). In general, as the microwave treatment period is increased, the ability of the protein to bind oil increases initially, then decreases. Protein reaggregation and a decrease in hydrophobic and non-polar amino acids are to blame for the dip. When red bean, wheat, and melanin wheat flour were heated in the microwave, for example, OBC grew at first, then reduced. At the same time, due to the variability of protein oil solubility, the oil absorption capacity of proteins varies.

#### Emulsification

Proteins' ability to diffuse over the oil-water interface and interconnect with water and hydrophilic amino acids, as well as oil and hydrophobic amino acids, is referred to as emulsifying capacity. Because the emulsion can change the food system to make it the ideal food, its development and stability during processing is critical. Protein emulsification is generated by the interaction of water and oil with the protein. The creation of these chemicals is beneficial to the food system and has an impact on the final product's flavor and texture. Microwave heating can cause the protein to unfold, exposing the hydrophobic unit. The unfolded protein then interacts with nonpolar solvents, preventing oil droplets from flocculating and improving the emulsion's overall stability. For example, microwave vacuum treatment (50–100 W mV) enhanced the stability of emulsion flocculation and paste of lotus seed protein isolate (LSPIs) (63).

#### Gel

During microwave heating, the protein conformational changes and subsequent intermolecular interaction are usually followed by stiffening and thickening of the pre-formed gel through thiol-disulfide exchange reactions (100). On the one hand, microwave treatment's high temperature may hasten the oxidation of protein sulfhydryl groups and increase disulfide bond content (80). Proteins then cross-link to form dense protein networks, which improves the gel's properties. Microwaves, on the other hand, increase exposure to the reactive phenol and mercaptan groups required to produce protein gels, which are normally embedded in natural proteins' dense structure (71). For example, acid gels formed with microwave treated casein solutions were harder and had a more compact microstructure compared to those made with heated caseins (51). In general, two types of protein gels are distinguished. When proteins with a high level of non-polar amino acid residues undergo a denaturation treatment, random aggregation rapidly takes place and an opaque coagulum-type gel is formed that usually contains covalent cross-links. Such protein denaturation and subsequent aggregation is irreversible. Proteins with fewer non-polar residues upon denaturation tend to engage in intermolecular interactions more gradually which results in ordered translucent gels. Here, upon removal of the denaturing conditions, proteins may to a degree refold. In a word, after microwave processing, enhanced protein gelation can increase food transparency and modify the taste and appearance of the final product.

#### Foam

Some scattered proteins and peptides have typical amphiphilic architectures that reduce surface tension and facilitate interface formation and foaming at the water-air interface. Because proteins and their hydrolysates quickly diffuse into the air-water interface and partially unfold to form a thin film with viscosity and flexibility, they are ideal foaming agents. Foaming capacity depends on the diffusion of protein at the air-water interface by unfolding its structure, while foaming stability is dependent on the formation of a thick cohesive layer around the air bubble (100). Partial enzymatic hydrolysis usually can improve foaming properties. The number of hydrophobic amino acids and substrates exposed to the surface of a protein molecule is directly proportional to its foaming capacity. Microwave-induced increases in hydrophobic amino acids increase protein viscosity and drive the formation of multilayer sticky protein films at the bubble interface, resulting in anti-coalescence. A thick membrane can limit the flow of proteins out of the membrane while also keeping the bubbles stable. Microwave-treated rainbow trout skeletal protein hydrolysates, for example, foamed better than enzymatic proteins (7). Rice bran protein treated by microwave had better foaming property than conventional heating (100). Many meals, such as cake, bread, and ice cream, require the foaming qualities of protein in daily life. The change of mechanical properties and the increase of gel properties after microwave processing can increase the transparency of food and change the taste and appearance of the final product, such as konjak, tofu and noodles. On the other side, it produces a more flexible and edible food packaging material. The quality of protein gelatin produced *via* microwave processing, however, remains uncontrolled. Microwave inhomogeneity can induce faults in the gel matrix, and too much power can cause the protein to expand too quickly, faster than the rate of polymerization, resulting in a rough gel network.

### Composition of amino acids

The total content of amino acids falls with microwave cooking, however the content of essential amino acids somewhat increases. Different types of amino acids have different variations in the microwave heating process. Because of heat intolerance or the Maillard reaction, the majority of hydrophobic and sulfur-containing amino acids increase, while a small number of amino acids, such as histidine and lysine, decrease. Aside from that, cysteine's contents don't alter significantly due to its heat resistance. Of course, the variation trends of the same amino acid in different foods are different, which could be connected to food type and microwave processing settings. In order to minimize the massive loss of heat-resistant amino acid components caused by long-term heating, special attention should be paid to the temperature, power, and duration in the microwave processing process for meals containing a large number of heat-resistant amino acids. The changes of different amino acid contents in food after microwave heating are shown in Table 7.

**Table 7:** Changes of different amino acid contents in food after microwave heating.

**Food species**	**Changes in amino acid content**	**References**
Lentils	Leucine goes up and valine stays the same	(102)
Soy milk	Lysine, tryptophan and cysteine decreased	(103)
Buckwheat	Serine, proline, leucine and phenylalanine increased, methionine and tyrosine did not change significantly, but some sensitive amino acids, such as histidine, are reduced	(51)
Sunflower	Proline, aspartic acid, threonine, valine, isoleucine and lysine increased	(104)
Fish fillet	Lysine, valine, alanine and leucine increased slightly, aspartate, threonine and cysteine changed little, but histidine decreased	(52)
Rice	Glutamic acid, glycine, alanine, phenylalanine, valine, lysine, methionine, leucine, isoleucine increased, serine changed little, Aspartic acid, arginine, cysteine, but Aspartic acid arginine cysteine histidine and tyrosine decreased	(74)
Milk protein concentrate	Cysteine, valine, phenylalanine and tyrosine increased, while alanine, leucine and proline decreased	(86)

## The influence of microwave on flavor, nutrients and security

### Flavor

Alcohols, ketones, hydrocarbons, lipids, organic acids, heterocyclic compounds, free amino acids, and other components in food alter the flavor of the dish. Aldehydes, for example, have a volatile and greasy fragrance (105). Pelargonic aldehyde smells like roses (106). Sweet amino acids include glycine, alanine, serine, threonine, and proline, while bitter amino acids include methionine, leucine, isoleucine, histidine, arginine, and phenylalanine (84).

The synthesis and adsorption of taste compounds are the key reasons for the improvement of flavor in food cooked in the microwave. On the one hand, taste compounds can be produced through the microwave-promoted Maillard process, lipid oxidative degradation, and protein hydrolysis. Alcohols and organic acids, for example, are produced *via* oxidative lipid degradation. The Maillard process produces pyrrole, pyrazine, and sulfur compounds (107). Microwave, on the other hand, changes the structure of proteins and increases the number of binding sites that react with volatile chemicals, boosting taste adsorption (1, 108). Microwave cooking has been shown to improve protein adsorption to ketone flavor compounds by increasing the concentration of sulfhydryl groups, which can react with the ketone and produce disulfide (109). Microwave protein has a better binding affinity to ketone taste chemicals than conventional water cooking heating which just affects the surface hydrophobicity (1). The umami flavor of mushrooms can be enhanced by increasing the concentration of aspartic acid and glutamic acid in the microwave. It also decreased the content of organic acids in the product to reduce astringency. Although the amount of mannitol, fructose, and trehalose in the solution was lowered, the retention rate was higher than with ordinary heating (110). Furthermore some undesirable flavors can also be reduce by cross-linking (111). Microwave heating, on the other hand, should be avoided to take a long time or a high power. Because protein aggregation can cause the sulfhydryl to become buried inside the protein, reducing its ability to bind to flavor compounds (112, 113).

### Nutrients

Small molecular components, minerals, and vitamins are the main nutrients in food, in addition to protein, carbohydrates, and lipids. Although the effect of microwave cooking on vitamins varies, it often outperforms traditional cooking methods like as boiling. Due to the water avoidance and shortening of treatment time in this process, microwave treatment can prevent the loss of vitamins A and C owing to water and reduce the thermal degradation of vitamins B1 and B6 (114). Microwave cooking, for example, had a retention rate of carotene that was 1.31–1.83 times higher than conventional water cooking (115). The most easily degraded form of vitamin E is α-tocopherol, but microwave-induced total disintegration of plant cell walls could improve α-tocopherol extractability and hence increase its amount. Cooking fresh broccoli, Swiss chard, mallow, daisies, Perilla leaves, spinach, and zucchini in the microwave, for example, resulted in a considerable rise in α-tocopherol (60). Some oxidases, which can be activated by plant tissue damage induced by cutting or mixing, may be involved in the effect of microwave on vitamin E levels. Microwave heat treatment can inactivate natural oxidases, enhancing vitamin E retention. Because of vitamin K's thermal stability, microwave heating has no effect on it. Microwaves can also preserve the mineral content of food. The quantities of Na, K, and P in raw trout, for example, increased considerably following microwave heating (7).

### Security

It's been a common misconception that microwaved food might cause carcinogens. According to studies, microwave heating not only prevents the formation of heterocyclic amines and other carcinogens, but also regulate the allergenicity of proteins, reduce the accumulation of saturated fatty acids and trans fatty acids, which lowers the risk of allergic reactions and cardiovascular illnesses. Heterocyclic amines (HCAS) are mutagenic, carcinogenic, and cardiotoxic chemicals formed when protein amino acids are pyrolyzed during food preparation (116). Microwave can inhibit the production of heterocyclic amines, which may be related to its effect on the amino acid composition of protein and the improvement of the antioxidant capacity of some components. First of all, microwave can regulate the composition of amino acids in food proteins, and proline and other essential amino acids have been proven to reduce the production of heterocyclic amines by inhibiting the production of heterocyclic amine intermediates or precursors, forming adducts with heterocyclic amines and heterocyclic amine intermediates (117). Secondly, microwave improves the antioxidation of some food ingredients, which can prevent the free radical chain reaction that produces heterocyclic amine, hence lowering the production of heterocyclic amine (118). Finally, the temperature and duration are the most important variables in the production of dioxins, heterocyclic amines, and other chemicals. Foods high in fat can easily produce these compounds through prolonged high-temperature cooking. Therefore, Therefore, one of the factors contributing to the reduction of heterocyclic amines is the microwave's short-time and low-temperature cooking features. For example, Microwave pretreatment of fried beef pie could reduce fat, water, and HCAS precursors, compared with conventional heating (119).

In addition, microwaves help prevent food from becoming contaminated with microorganisms. Aflatoxin in naturally contaminated peanuts can be efficiently destroyed by microwave baking in the range of 32–40%. Beverage items frequently mildew and contain high amounts of bacteria, and is unsuitable for sterilizing at high temperatures (120). Given these considerations, using microwave technology for sterilizing at a low temperature and quick speed is an excellent solution, since it not only kills all types of bacteria in the drink but also prevents mildew throughout the storage process.

## Conclusion

In this manuscript, we have found that microwave heating can change the structure and specific qualities of starch, lipid, and protein in food, making it an excellent food to meet the new needs of markets. For particular properties: (1) For the puffy products with toughness and brittleness: Microwave can improve the gel property of protein and reduce the viscosity, expansion, and gelatinization of starch to meet these goals. (2) For products that need foam, such as cake, bread and ice cream: Microwave can help produce the desired results. (3) For the low sugar products that diabetics and dieters require: Microwave can increase the resistant starch content to reach these targets. (4) For the easily oxidized products: Microwave can increase the antioxidant activity of some components and reduce body damage caused by peroxidation. (5) For emulsified products: Microwave can increase the stability of emulsified food and prevent the flocculation of ingredients. (6) For persons who have specific amino acid requirements: The microwave can boost the quantity of certain specific amino acids in food. (7) For allergy sufferers: Microwave can reduce the allergenicity of their meals. At the same time, there are no safety concerns with microwave-processed foods, and more flavor and small molecular nutrients such as vitamins and minerals have been preserved. These evidences demonstrate that the microwave can replace traditional food processing processes.

However, microwave heating is still insufficient, such as (1) The complex composition of the foods influences the final qualities of foods during microwave treatment. Some foods will become more allergic after being microwaved. Therefore, due to the unknown nature of food, it is necessary to carry out strict process screening before processing. (2) When the microwave process parameters do not match the food, the quality of the finished product will be completely different from the expected. As a result, while utilizing microwaves for food processing, we must consider the meal's composition, heating power, time, temperature, etc. In this way, it can play its greatest advantage in food processing. Furthermore, the majority of available research focuses on the effect of microwaves on single food components, with few investigations involving numerous components at the same time. However, it is not difficult to deduce from this paper that if you want to fully exploit the performance advantages of various food Components, you must select appropriate microwave parameters based on the requirements of target products and the change law of component properties, but different components have different or even opposite microwave parameter requirements. High microwave power, for example, promotes the synthesis of resistant starch but not protein digestibility, foaming, emulsification, or food flavor. At the same time, there are interactions among food components that impact the structure, properties, and functions of food in an indirect manner. As a result, in future related study, it will be important to take into account all of the different components in food and find the most appropriate processing procedure. It can also be regarded to affect the final nutritional qualities, structure, and texture of food by adding some components based on the interaction of starch, fat, and protein under microwave conditions.

## Author contributions

XD, HH, and YH contributed to conception and design of the study. XD and HH wrote the first draft of the manuscript. SH wrote sections of the manuscript. JW, ZC, and MY reviewed the manuscript. LH, DZ, and ZW reviewed and supervised the manuscript. All authors contributed to manuscript revision, read, and approved the submitted version.

## Funding

This work was supported by grants from the National Natural Science Foundation of China (82173991, 81760716, and 81960718), Open Project of Key Laboratory of Modern Preparation of Chinese Medicine (TCM-201904), Open Project of State key Laboratory of Innovation Medicine and High Efficiency and Energy Saving Pharmaceutical Equipment in Jiangxi University of Traditional Chinese Medicine (GZSYS202003), and National Interdisciplinary Innovation Team of Traditional Chinese Medicine (ZYYCXTD-D-202209).

## Conflict of interest

Author JW was employed by Xinqi Microwave Co., Ltd. The remaining authors declare that the research was conducted in the absence of any commercial or financial relationships that could be construed as a potential conflict of interest.

## Publisher's note

All claims expressed in this article are solely those of the authors and do not necessarily represent those of their affiliated organizations, or those of the publisher, the editors and the reviewers. Any product that may be evaluated in this article, or claim that may be made by its manufacturer, is not guaranteed or endorsed by the publisher.
